# Probing Chemical Changes in Holocellulose and Lignin of Timbers in Ancient Buildings

**DOI:** 10.3390/polym11050809

**Published:** 2019-05-06

**Authors:** Chencheng Zhao, Xiaochun Zhang, Lina Liu, Youming Yu, Wei Zheng, Pingan Song

**Affiliations:** 1 School of Engineering, Zhejiang A& F University, Hangzhou 311300, China; chenchengzhao0918@163.com (C.Z.); 20110039@zafu.edu.cn (X.Z.); liulina198310@126.com (L.L.); 2 Jiyang College, Zhejiang A& F University, Zhuji 311800, China

**Keywords:** aged timbers, ancient building, chemical analysis, structure evolution, morphology

## Abstract

Wooden structures in China’s ancient buildings hold highly historical and cultural values. There is an urgent need to repair and replace the damaged wooden structures after hundreds and thousands of years of exposure to weather. Unfortunately, to date there is still a lack of insightful understanding on how the chemical structure, composition, and micro-morphology evolve over the long-term natural aging before artificial ancient timbers can be developed. This work aims to systematically examine the outer surface, middle layer, and inner surface of the same piece of Chinese fir (*Cunninghamia lanceolate*) collected from an ancient Chinese building. Based on qualitative and quantitative analysis, both cellulose and hemicellulose in aged woods are found to experience significant degrees of degradation. The crystalline regions of cellulose are also determined to undergo moderate degradation as compared to the control fresh wood. In comparison, the lignin basically remains unchanged and its content in the inner layer slightly increases, as evidenced by more free phenol groups determined. Relative to the outer and inner layer, the middle layer of the ancient wood shows the lowest degree of degradation close to that of the fresh wood. This work offers guidelines for fabricating artificial ancient woods to repair the destroyed ones in China’s ancient architectures.

## 1. Introduction

**Figure 1:**
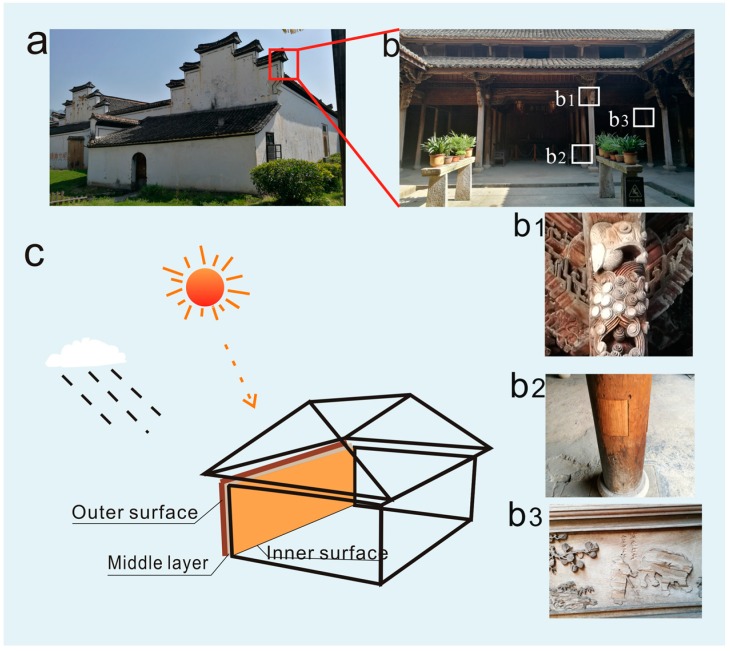
Damage and reparation of typical ancient wood buildings (**a**: ancient wooden building exterior view; **b**: ancient wooden building interior view; **b1**&**b2**: damaged wood carving; and **b3**: repaired with fresh wood; **c**: The specific positions for taking samples).

Wood culture is one of the most cultural heritages from ancient China, which has been handed down from generation to generation. In ancient China, most residences and temples are made of wood. As shown in Figure 1, wooden objects from old civilizations are considered precious and require careful storage and research, because they are susceptible to fungi, bacteria, and insects. As a result, after many years a vast majority of wooden structures in ancient buildings are significantly damaged, leaving only a small portion remains intact. The damage to the timbers in ancient buildings are irreversible and thus reparation and/or replacement are generally required to protect these historic ancient buildings. With the increasing awareness of protection of historical relics, the reparation of ancient architecture has become necessary. In order to develop efficient antique treatments to fresh woods, it is essential to gain an insightful understanding on the evolution of chemical/composition changes of the wooden structures during natural ageing [1].

Recently, the research on natural aging of wood has attracted extensive attentions. Carmen et al. [2,3,4] studied wood with different natural aging time and the results found that the proportion of carbohydrate gradually decreases, and the content of lignin increases successively with increasing time. This is due to the degradation of hemicellulose, which was mainly manifested in the decomposition of polyxylan. Michel et al. employed the solid-state nuclear magnetic resonance (SSNMR) to analyze the medieval canoe immersed by water and found that polysaccharides significantly degraded and hemicellulose almost completely degraded, however, the β-O-4 key of lignin remained [5]. Maria et al. detected the existence of free phenols from archaeological waterlogged wood [1], whereas Anna et al. investigated the physico-chemical characteristics of oak and Pinus densiflora being submerged by water [6]. Both researchers provided the theoretical basis for the studies on the degradation mechanism of lignin. Tamburini et al. pyrolyzed archaeological waterlogged wood followed by analyzing using mass spectroscopy (MS). The results show that polysaccharides degraded significantly and the relative content of lignin was increased [7]. Colombini et al. found that most of the lignin was acetylated, the molecular weight of lignin increased, and the phenmethyl groups near to the β-O-4 bond were oxidized [8].

Generally, cellulose, hemicellulose, and lignin in wood undergo different extent of change during natural aging. The changes of three components have been widely examined in the aging process of wood. Lucejko JJ et al. showed that the degradation of fiber bundles and other sugars were catalyzed in water but the overall structural changes of lignin did not occur [9]. In all waterlogged ancient wood samples, β-O-4 chains were not destroyed. Saiz-Jimenez C et al. [10] reported that the degradation of lignin was more difficult than other components and only a small percentage of lignin is decomposed by fungi and bacteria. In water environments, microbial is the main factor of wooden degradation, which is why some wood antiquities, such as shipwrecks, are easier to preserve. Karami L et al. studied the degradation of naturally aging oak bridge by using pyrolysis gas chromatography-mass spectrometry (Py-GC/MS) and the ratio of lignin and carbohydrate was found to increase, whereas S-type and G-type lignin units decrease, reflecting the different degree of degradation of different components in the aging process [11]. The study on the degradation of lemon murals relics showed that the degradation of cellulose and hemicellulose caused the increased relative content of lignin [3]. Previous research has clearly demonstrated that both cellulose and hemicellulose experienced a great extent of degradation under light oxidation and microbial erosion, but the lignin remained intact in the process of natural aging. Despite great advances, there remains a lack of insightful understanding on how the chemical structure, composition and micromorphology of timbers in ancient buildings evolve during ageing.

This work aims to qualitatively and quantitatively probe the evolution of the chemical structure, relative content and micromorphology of timber in China’s typical ancient buildings. Various analyses including Fourier transform infrared spectroscopy (FT-IR), Py-GC/MS, X-ray diffraction (XRD), X-ray photoelectron spectra (XPS), wood staining, scanning electron microscopy (SEM), and thermogravimetry (TG) are employed to determine the difference between fresh wood and ancient wood. This work provides valuable guidelines for creating artificial ancient woods to repair and replace the destroyed ones in China’s ancient architecture.

## 2. Materials and Methods

### 2.1. Materials

Ancient wood samples were Chinese fir (*Cunninghamia lanceolata*) taken from an about 170 years old wooden building in Lishui City, Zhejiang Province, China. The wood samples were tagged as LS-M (samples from the half of the thickness of the wood), LS-I (the innermost layer of wood with a thickness of about 1 mm), and LS-O (the outermost layer of wood with a thickness of about 1 mm), respectively (Figure 1). The control sample (CS) was fresh wood with the same tree species of ancient wood samples. The specific positions for taking samples have been indicated in Figure 1c. The original materials were crushed and pulverized to a size of <0.2 mm, and oven-dried at 105 °C before they were analyzed.

### 2.2. Determination of Lignin, Cellulose, and Hemicellulose

The content of holocellulose of the samples was determined by dissolving the lignin in acidified sodium chlorite solution [12]. The content of cellulose was determined according to the National Standard of GB/T 2677.10-1995 and GB/T 2677.8-1994 after dissolving the hemicellulose from the holocellulose and the difference between holocellulose and cellulose was reported as the hemicellulose content. The content of lignin were determined according to National Standard of GB/T 744-1989. Briefly, samples were first submitted to Soxhlet extraction with ethanol/toluene (1:2, v:v) [13] and then treated with 72 wt.%. sulfuric acid to dissolve cellulose, hemicellulose and acid soluble lignin [14].

### 2.3. Characterizations

Fourier transform infrared spectrometer (NICOLET IMPACT410, NICOLET, Madison, WI, USA) was used to identify the functional groups of the components. The samples are produced by the potassium bromide tableting method with a scanning wavenumber range of 400–4000 cm^−1^, and the number of scanning times is 32.

Py-GC/MS has been applied to evaluate the thermal decomposition behavior and to determine the pyrolysis products of polymers due to its rapidity, high sensitivity, and effective separation of complex mixed compounds [15,16,17,18]. Pyroprobe analytical pyrolyzer (CDS 5200, Chemical Data Systems, CDS Analytical, Oxford, PA, USA) in combination with GC/MS (Agilent Technologies 7890B / 5977B, Agilent Technologies, Palo alto, CA, USA) to investigate the distribution of lignin fast pyrolysis products. Approximately 0.5 mg of sample was fed into the quartz pyrolysis tube. The pyrolysis temperature was set to rise from room temperature to 800 °C, the fixed heating rate was 20 °C /ms, the fixed residence time was 20 s, and the transmission line and injector temperature were maintained at 300 °C and 300 °C, respectively. Chromatography was carried out by using an HP-5MS capillary column (inner diameter: 30 m × 0.25 mm × film thickness: 0.25 µm). The carrier gas was helium (99.999%) with a constant flow rate of 3 mL/min, and the split ratio was 1:100. The furnace temperature was set from 40 °C (for 3 min) to 280 °C (for 3 min) with a heating rate was 10 °C /min. The mass spectra were obtained when the mass to charge ratio (*m*/*z*) was 50–400 in electron impact ion source (EI) mode of 70 eV. Based on the National Institute of Science and Technology (NIST) library and related literatures, the organic evolutionary components corresponding to each chromatographic peak can be identified in the Mass Hunter workstation software. It has been proved that the absolute peak area can be used for the quantitative determination of aromatic compounds.

The XRD tests were carried out on a DZF-6210 (Shimadzu Corporation, Kyoto, Japan) X-ray diffractometer. The sample (60–100 mesh) was pressed into thin slices at room temperature. The intensity was measured as a function of the scattering angle 2*θ* by *θ*-2*θ* scan. The angle range was 5–35° and the scanning speed is 2 °/min. A degree of crystallinity CRx was determined by the Segal method [19], using the height of the (200) peak (*I*_200_, 2*θ* = 22.7°), and the minimum between the (200) and (110) peaks (*I*_AM_, 2*θ* = 18°) by the following equation:

$${\mathrm{CR}}_{X}=\frac{I_{200}-I_{\mathrm{AM}}}{I_{200}}*100\%$$

*I*_200_ represents both crystalline and amorphous material and *I*_AM_ represents only amorphous material.

Thermogravimetric analysis (TGA) tests were performed on a NETZSCH STA409C (NETZSCH, Selb, German) thermogravimetric analyzer. Typically, about 8.0 mg of sample was heated from 30 °C to 800 °C at a heating rate of 10 °C /min under nitrogen atmosphere.

Elemental analysis was performed on a Vario EL cube (elementar company, Frankfurt, German) automatic analyzer. Based on its Duma thermal decomposition means and dynamic gas separation technology (adsorption analysis principle), as well as the brilliant detecting methods. In order to achieve high accuracy, we measured a sample with a wide range of size distributions and concentrations. During the test, the temperature of the reducing pipe was 850 °C, the burning pipe was 1150 °C and the pressures of nitrogen and oxygen were 0.15 MPa and 0.20 MPa, respectively. Sulfonamide standard substance is 5.0 mg and the sample weight is 4.8–5.2 mg, with 90 s oxygen-adding time.

Scanning electron microscope (SEM, TM3030, Hitachi High-Technologies, Tokyo, Japan) was used to observe the section topography of sample at an accelerating voltage of 5 kV. The sample was cut into slices about 80 μm thick then dry at 105 °C for 2 h.

The X-ray photoelectron spectroscopy (XPS, Perkin Elmer, Waltham, MA, USA) analyses were carried out with a PHI-5400 electron spectrometer and using a monochromatic Al Kα source (1486.6 eV). Survey spectra were recorded in 1.0 eV steps and 200 eV analyzer pass energy. The samples were prepared with a thoroughly cleaned and vacuum drying.

## 3. Results and Discussion

### 3.1. Determination of Lignin, Cellulose, and Hemicellulose

The change in Klason lignin is opposite to that in the carbohydrate portions and extractive compounds. As listed in Table 1, the content of lignin in CS is about 31.37%, which is lower than that of the samples LS-M (34.00%) and LS-I (34.51%), but higher than that of LS-O (30.05%). This may be attributed to the chemical degradation of cellulose, hemicellulose, inorganic elements and extractive, resulting in the increased relative content of lignin [20]. Another reason for this could be the tree used to build the structure had a higher lignin content to begin with. In comparison, because the sample LS-O is exposed to the sun and ultraviolet light longer than both LS-M and LS-I, the lignin degrades more, thus showing the least content of Klason lignin.

### 3.2. FTIR Analysis

**Figure 2:**
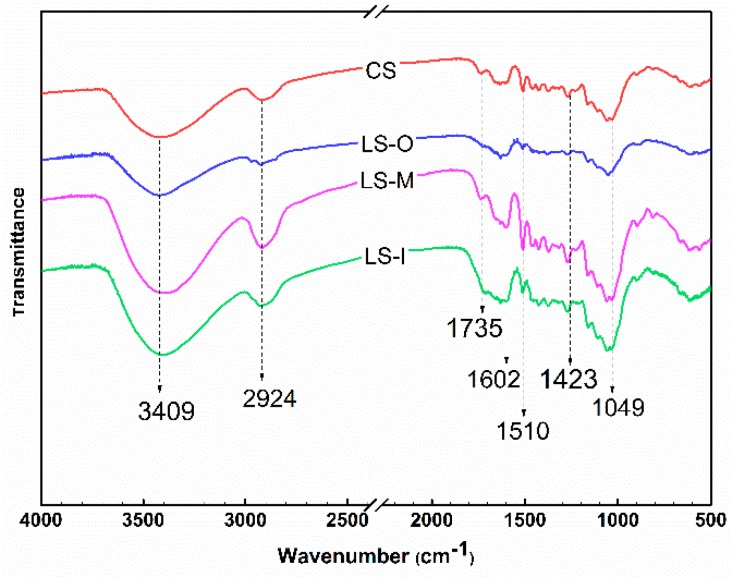
Infrared spectra of CS, LS-O, LS-M and LS-I.

As shown in Figure 2, the 3409 cm^−1^ band is ascribed to the stretching vibration of hydroxyl groups (–OH) [21,22,23,24]. The sample LS-O has the lowest intensity followed by sample LS-I, which indicated the occurrence of dehydration during natural ageing. Compared with the fresh wood, the stretching vibration peak (2924 cm^−1^) of methyl(–CH_3_) and methylene (–CH_2_–) [25] in three samples of natural aging wood becomes flat and the LS-O shows the smallest absorption intensity. The peaks in FTIR at 1735 cm^−1^ were due to the C=O stretching vibration in xylan [26]. Interestingly, the ancient wood sample does not show an absorption peak at around 1735 cm^−1^ belonging to the stretching vibration of the carbon-oxygen double bond in acetyl (CH_3_C=O) group of xylan, indicating a very low content of hemicellulose in the ancient wood sample. The stretching vibration located at 1602 cm^−1^ of the conjugated carbonyl in the side chain of the lignin [26] structural unit shows that the characteristic peak of the natural aged fir is still obvious. This means that the lignin only slightly degrades. The signals at 1510 and 1423 cm^−1^ correspond to the aromatic skeletal vibrations and the aromatic skeletal vibrations combined with C-H in-plane deformation [27], respectively. LS-O has the lowest intensity followed by sample LS-I and CS whereas the LS-M has the highest intensity due to the degradation of the aromatic structures. This is consistent with the previous determination of Klason lignin content.

### 3.3. Py-GC/MS

**Figure 3:**
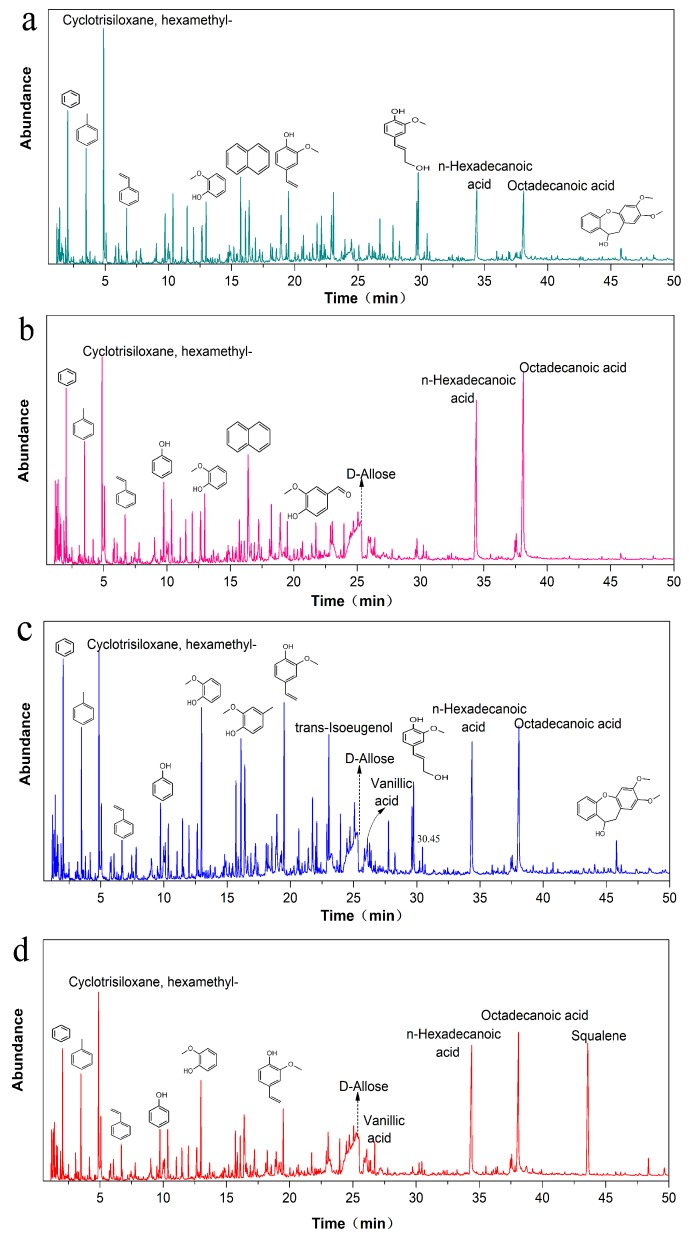
Detailed Py-GC/MS data for (**a**) CS, (**b**) LS-O, (**c**) LS-M, and (**d**) LS-I.

Furans compounds are used to analyze the fresh Chinese fir, and furfural compounds derive from cellulose and hemicellulose whereas aromatic compounds are from lignin. Figure 3 shows the detailed data for the pyrolysis products from Chinese fir and aged Chinese fir, with each peak corresponding to one specific compound. It is found that the peaks of Chinese fir are basically similar to the aged one, indicating that ageing does not change the chemical compositions of the pyrolysis products. The peaks between a Retention time (RT) from 9.73 to 28.27 (Tables S1–S4) belong to aromatic compounds corresponding to the intensity peak. As compared with the fresh wood, ageing Chinese fir also shows the peak for aromatic compounds but the intensity does not reduce. Aromatic compounds are produced by the rapid pyrolysis of lignin, proving that the benzene ring structure of lignin is not destroyed or changed significantly after natural aging due to the chemically stable six-membered ring symmetry structure of the benzene ring. The peaks at a RT range below 9.01 are ascribed to carbohydrate compounds and the peak intensity of naturally aged one less than nine is much weaker than that of fresh wood. Because these carbohydrates are mainly produced by the thermal decomposition of cellulose and hemicellulose, therefore some cellulose and hemicellulose of natural aging fir are decomposed and lost during natural ageing. More importantly, the pyrolysis peaks at RT = 12.98, 17.4, 19.5, and 23.04, respectively, correspond to guaiacol, 4-methyl guaiacol, 2-methoxy-4-Vinylphenol, and trans-2-methoxy-4-o-propenyl phenol. They are primarily derived from guaiacol-based phenolic monomers in lignin, indicating that lignin in Chinese fir is mainly composed of guaiacol monomer.

The content of phenolic derivatives in the sample LS-I is higher than that of LS-O and LS-M. This is because the aging degree of the sample LS-I is higher than that of LS-M resulting in the increased relative content of lignin. However, the content of phenolic derivatives in LS-O is the lowest, because the content of lignin in LS-O is the lowest due to the sample was exposed to sunlight and air for a long time. As compared with the control fresh wood, the intensities of these peaks for naturally-aged Chinese fir pyrolysis compounds decrease. Unexpectedly, the content of phenolic derivatives in the aged wood samples is higher than that of fresh wood. Hence, the main difference between archaeological lignin and sound lignin may be that the archaeological lignin contains a relatively higher content of phenolic derivatives. Another reason for this could be the tree used to build the structure had a higher phenolic derivatives content to begin with.

**Figure 4:**
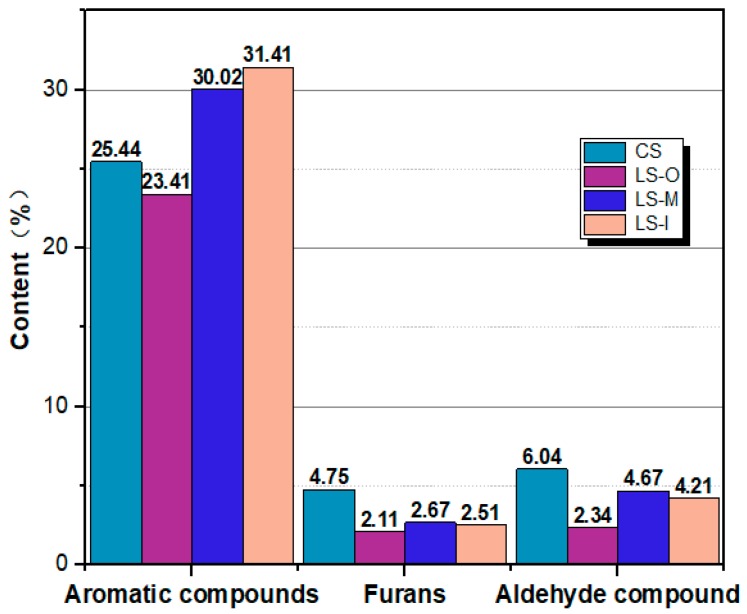
Main pyrolysis products contents for CS LS-O LS-M and LS-I obtained from Py-GC/MS measurements.

As shown in Figure 4 (The data in the figure is obtained according to Tables S1–S4), the pyrolyzed products of CS, LS-M, LS-I, and LS-O, respectively, exhibit 25.44%, 30.02%, 30.35%, and 23.41% of aromatic compounds. This is because of the significant degradation of both hemicellulose and cellulose whereas the lignin almost does not decompose in naturally aged fir, thereby leading to a decrease in the relative content of carbohydrates in the pyrolyzed products. As a result, the relative content of aromatic hydrocarbons in the pyrolysis products from ancient wood increases with the degree of aging.

### 3.4. Elemental Analysis

Cellulose mainly contains three kinds of elements, namely carbon, hydrogen, and oxygen, with a chemical composition of 44.2%, 6.3%, and 49.5%, respectively. In comparison, lignin is composed of 60.04% C, 5.98% H, and 31.29% O. As summarized in Table 2, compared to the LS-M the content of elements carbon and hydrogen in sample LS-O and LS-I decreases but the content of oxygen increases due to the more severe oxidation of lignin and cellulose occurred in the aging process. Compared with CS the content of elements carbon, hydrogen in naturally aged samples all decrease but the content of oxygen increase. This may be the result of the oxidation of cellulose, lignin and hemicellulose, but it can not exclude that it is caused by the tree used to build the structure had higher oxygen element content.

Meanwhile, the content of both elements N and S in ancient Chinese fir are obviously higher than that of the fresh wood and the LS-O shows the highest contents of N and S, followed by LS-M and then LS-I. This is because the timber in the buildings is seriously attacked by acid rains, which usually contains a certain content of SO_2_ and NO_2_ in ancient Chinese fir with long-term exposure to external environments. In this study, the ancient wood samples were taken from the windows of ancient buildings. In the process of natural aging, the sample LS-I was also affected by the flowing humid air containing H_2_SO_3_ (SO_2_ + H_2_O = H_2_SO_3_) when the windows are open sometimes, thus resulting in the increase of S content.

### 3.5. XRD

As presented in Table 3, there is no significant change observed in the peak shape for the four samples except for the intensity. The crystalline morphology of cellulose in Chinese fir is found to only slightly change after many years of natural ageing. The intensity of diffraction peaks for naturally aged fir is significantly increased, which is due to the significant loss of hemicellulose but slight loss of cellulose, thereby retaining the cellulose crystalline zone. It can be found that in Table 3 and Figure S1, the crystallinity of ancient wood decreases with respect to that of fresh wood. In addition, the crystallinity of ancient wood decreases in the order of LS-O < LS-I < LS-M, which is due to the loss of cellulose that leads to reduced crystallinity.

### 3.6. Thermal Analysis

Hemicellulose shows the lowest thermal stability in the wood components and starts to degrade around about 225 °C, with a rapid degradation temperature range of 210–320 °C. In contrast, cellulose is more thermally stable, showing a narrow thermal degradation temperature range between 300 and 375 °C. As compared with hemicellulose and cellulose, the aromatic structure makes lignin exhibit the highest thermal stability. Its thermal decomposition mainly takes place between 250–500 °C and decompose fastest between 310–420 °C [28,29]. Due to the differences in the structure, content and physical properties, these three major components demonstrate different pyrolysis characteristics but may mutually interfere in the thermal degradation process because of their strong interactions [30].

**Figure 5:**
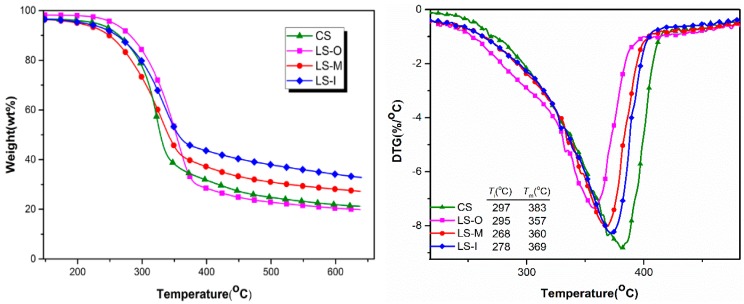
Thermal Gravity Analysis (TG)/Differential thermal gravity (DTG) analysis curves of different samples. Notes: Ti and Tm refer to the temperature where starting decomposition and the maximum mass loss occur.

As shown in Figure 5, there are no identified weight losses for four samples before 200 °C and the decomposition starts from 268–300 °C. The maximum weight loss peak of CS appears at about 383 °C and the weight loss process ends at about 400 °C, leaving a final residue of 19.89%. In the case of the aged wood, LS-M, LS-I, and LS-O, respectively, exhibit a maximum weight loss peak at 360 °C, 369 °C and 357 °C. The pyrolysis of both lignin and hemicellulose is mainly through the removal of side chains followed by the depolymerization of polymer chain and intramolecular dehydration. During the natural aging, lignin, and hemicellulose degrade to generate low molecular weight substances, resulting in increased lateral chains on hemicellulose and lignin. The increase of side chains leads to the decrease in the initial pyrolysis temperature and the temperature of rapid pyrolysis. LS-O shows the lowest maximum degradation temperature due to its lowest contents of lignin, cellulose, and hemicellulose [31].

LS-O, LS-M, and LS-I show a final residue mass of 21.73%, 27.21%, 32.75%, respectively. TG results show that the onset pyrolysis temperature of all the samples does not change significantly and all of them start to pyrolyze at 268–300 °C. Due to different contents of the three major components, the maximum weight loss peak has a slight difference. The LS-I was the highest residue, about 32.75%, while the difference in the other groups is just about 3–8%. This is due to the degradation of cellulose and the relative increase content of lignin, thus leading the wood sample to exhibiting high content of carbon.

### 3.7. Morphology Observation

**Figure 6:**
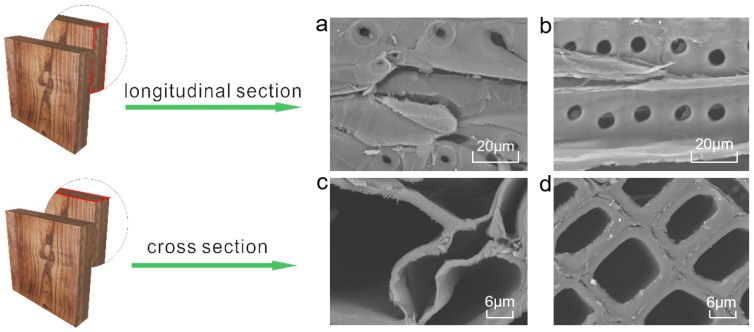
SEM images of the cross-section topography of (**a**) and (**c**) the outer layer of ancient timbers, (**b**) and (**d**) the control fresh samples.

**Figure 7:**
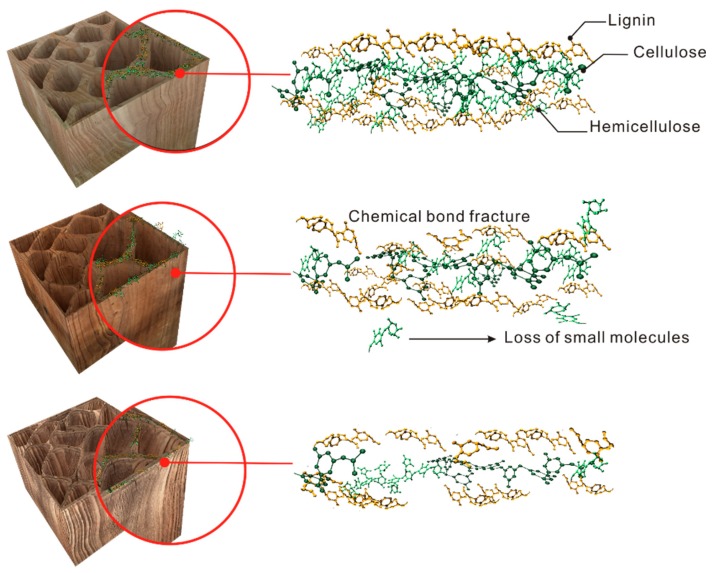
Schematic illustration of the change of three major components in the timber in the ancient buildings in the natural aging process.

Figure 6 clearly shows that the pith structure of fresh wood is integral whereas that of the ancient wood is deformed with some filamentous residue attached. At higher magnifications, we can observe the more intact xylem cells and intercellular layer structures. The cell wall of the sample LS-M slightly changes. In comparison, that of LS-I becomes thinner but the damage degree is less than that of LS-O (Figure S2). These are due to the large loss of cellulose and hemicellulose in aged Chinese fir, resulting in an increase in the intercellular space and a deformation of cell compartment well consistent with the results of FTIR and Py-GC/MS. Figure 7 illustrates the proposed process for the loss of cellulose and hemicellulose of timbers in buildings.

### 3.8. XPS

**Figure 8:**
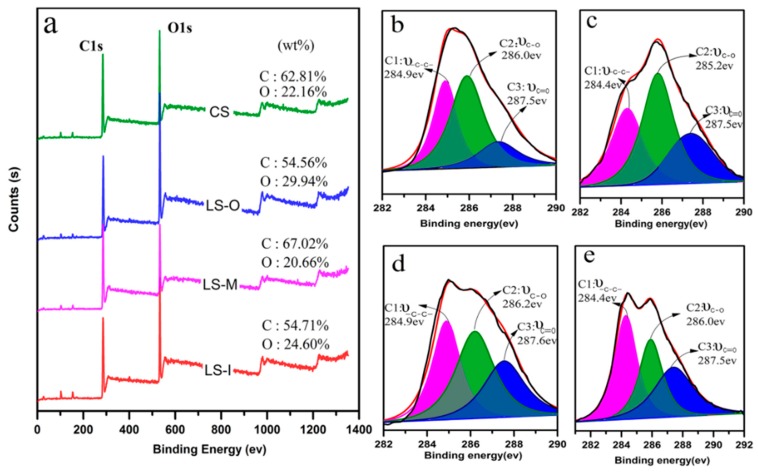
(**a**) X-ray photoelectron spectroscopy (XPS) survey spectra of four samples and C1s spectra of (**b**) CS, (**c**) LS-O, (**d**) LS-M, and (**e**) LS-I.

To gain an insightful understanding on the change of various functional groups, the C1s spectrum is usually deconvoluted into three components according to the number of oxygen atoms bonded to C. The C1 class at a binding energy (BE) of 284.6 eV corresponds to carbon atoms bonded only with carbon or hydrogen atoms. This peak mainly arises from lignin phenyl propane and fatty acids, fats and waxes and other hydrocarbons. The C2 class at a higher BE compared to C1 (Δ_BE_ = +1.5 ± 0.2 eV) reveals the carbon atoms bonded with one oxygen atom. A large number of carbon atoms in cellulose and hemicellulose molecules in wood are connected with hydroxyl (–OH). In comparison, the C3 class belongs to carbon atoms bonded to a carbonyl or two non-carbonyl oxygen atoms (Δ_BE_ = +2.8 ± 0.2 eV), representing the characteristic peaks of carbonyl groups and the oxidation in wood [2]. Figure 8a shows that LS-M gives rise to the highest C/O ratio relative to the reference sample and LS-I and LS-O because of the lowest oxidation degree. LS-O and LS-I exhibit a lower C/O ratio than the CS because the long-term exposure to the environment leads to oxidation. The considerable changes are observed on the sample LS-O and this variation can be explained by the removal of low molecular weight compounds as extractives, hemicelluloses and even a certain part of amorphous cellulose in the first period of ageing [32].

Based on the XPS results (Table 4 and Figure 8), we can calculate the proportion of three kinds of C1s peaks. The CS shows the largest C2 peak whereas the sample LS-I exhibits the smallest peak. In addition, the maximum and the minimum C3 peaks are observed in LS-O and CS, respectively. The C2 (C-O groups) peak area reflects the characteristic structure of cellulose and hemicellulose. This means that the CS sample contains the most cellulose and hemicellulose. Similarly, the C3 peak reflects the oxidation degree of the lignin component on the timber surface. In brief, the degrees of oxidation of four samples are different and the oxidation of LS-O is the most serious whereas the CS shows the lowest degree of oxidation. Moreover, both cellulose and hemicellulose suffer more oxidation than the lignin.

## 4. Conclusions

This work systematically investigates the changes of chemical structure and composition and morphology of timbers after many years of natural ageing in ancient Chinese buildings. Under the experimental conditions in this work, the inner layer of the ancient wood samples has the highest content of holocellulose, lignin and the crystallinity followed by the middle layer and then the outer layer. The content of aromatic compounds and aldehydes in pyrolysis products is also the highest in the middle layer and the dehydration of the outer layer is the most serious during wood aging. The outer layer of ancient wood undergoes the largest degradation as compared with the other two layers. Compared with the fresh wood samples, the ancient timbers show significant reductions in the content of cellulose and hemicellulose and their degrees of crystallinity. The crystalline zone of the cellulose in ancient wood samples is partially destroyed. The outer layer of the ancient timbers shows the higher degree of oxidation than the other two layers and the control of fresh wood due to long-term exposure to various environments. During natural ageing, both cellulose and hemicellulose loss leaving deformed pith structures whereas the chemical structure of lignin remains intact except for the outer layer due to long-term exposure to ultraviolet light. In brief, the outer layer of wood exhibits the most serious ageing, while the middle layer only undergoes slight ageing. This work offers an insightful understanding of the evolution of physical features of the ancient timber during a long-term natural ageing and thus provides guidelines for producing artificial ancient woods to repair the damaged ones in ancient Chinese architecture.

## Figures and Tables

**Table 1:** The content (wt %) of lignin (Klason lignin), cellulose, hemicellulose, and holocellulose, for the archaeological and fresh wood examined in this study.

Composition	CS	LS-O	LS-M	LS-I
Klason lignin (wt %)	31.37	30.05	34.00	34.51
Holocellulose (wt %)	66.85	56.65	63.33	59.42
Cellulose (wt %)	46.53	41.87	45.29	43.63
Hemicellulose (wt %)	20.32	14.78	18.04	15.79

**Table 2:** The elemental analysis results of the CS, LS-O, LS-M, and LS-I.

Sample	C (wt %)	H (wt %)	O ^1^ (wt %)	N (wt %)	S (wt %)
CS	48.79	6.90	44.29	0	0.018
LS-M	47.83	6.75	44.62	0.64	0.161
LS-I	47.52	6.67	45.10	0.62	0.094
LS-O	47.24	6.53	45.31	0.76	0.164

^1^ The content of oxygen (O) is based on relative contents of elements C, H, N, and S.

**Table 3:** The degree of crystallinity of different samples.

Sample Number	CS	LS-O	LS-M	LS-I
Degree of crystallinity (%)	35.89	32.04	35.81	34.62

**Table 4:** Carbon peak area ratios for C1s binding energy for CS, LS-O, LS-M, and LS-I.

Sample	Peak Area Percentage (%)		
	C1 (C–C)	C2 (C–O)	C3 (C=O)
CS	34	50	16
LS-O	31	35	34
LS-M	33	40	27
LS-I	38	30	32

## Supplementary Materials

The following are available online at https://www.mdpi.com/2073-4360/11/5/809/s1, Figure S1: XRD spectra for CS, LS-I, LS-O, and LS-M. Figure S2: SEM images of cross-section topography of ancient timbers, (**a**) LS-M and (**b**) LS-I. Table S1: The detailed results of PY-GC/MS test of samples at the RT range of 1–10 min. Table S2: The detailed results of PY-GC/MS test of samples at the RT range of 10–20 min. Table S3: The detailed results of PY-GC/MS test of samples at the RT range of 20–30 min. Table S4: The detailed results of PY-GC/MS test of samples at the RT range of 30–40 min.
