# Supplementary material for: Probing Chemical Changes in Holocellulose and Lignin of Timbers in Ancient Buildings
Source: Polymers (Basel). 2019 May 6;11(5):809. doi: 10.3390/polym11050809 (PMC6572151; doi:10.3390/polym11050809)
Supplement: Supplementary file 1 [file polymers-11-00809-s001.pdf]

Supporting Information

Probing Chemical Changes in Holocellulose and Lignin of Timbers in Ancient Buildings

Chencheng Zhao [1, #], Xiaochun Zhang[1, #], Lina Liu [a], Youming Yu [1,*], Wei Zhen [2,*], Pingan Song [1,*]

[1] School of Engineering, Zhejiang A& F University, Hangzhou 311300, China

[2] Jiyang College, Zhejiang A& F University, Zhuji 311800, China

[#] Both authors contributed equally to the work and serve as co-first authors.

*Correspondence should be addressed at: yuyouming@zafu.edu.cn; 415972228@qq.com, pingansong@gmail.com

Table of Contents

Figure S1

Figure S2

Table S1-S4

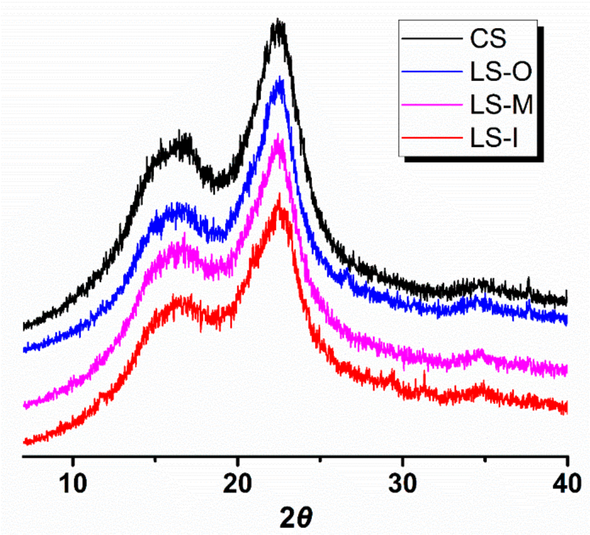

Figure S1. XRD spectra for CS, LS-I, LS-O and LS-M.

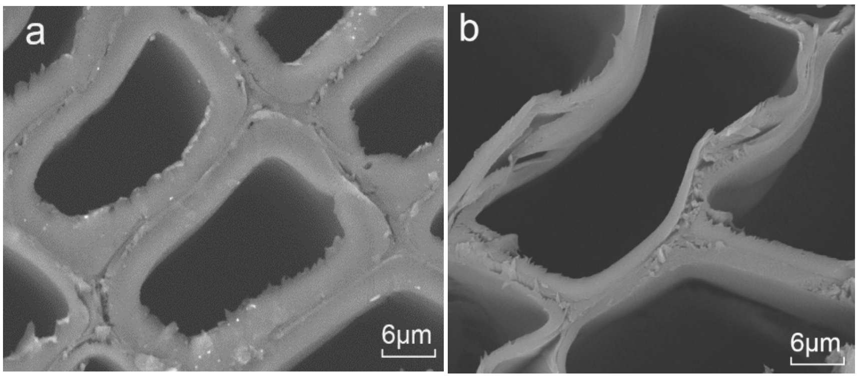

Figure S2 SEM images of cross-section topography of ancient timbers, a) LS-M and b) LS-I

Table S1. The detailed results of PY-GC/MS test of samples at the RT range of 1-10 min.

RT/min	Compound	Peak area percentage (%)			
		CS	LS-M	LS-I	LS-O
1.13	Sulfur dioxide				1.1
1.17	1,3-Butadiene	0.65	0.64	0.77	
1.27	2-Propenal		0.38	0.74	1.01
1.31	1,3-Pentadiene	0.45	0.44		0.89
1.40	1,3-Cyclopentadiene	0.76		0.65	1.03
1.49	Methacrolein	0.36			
1.63	Furan, 3-methyl-	0.41	0.19	0.45	0.85
2.03	Benzene	3.16	2.54	2.17	2.61
2.50	Furan, 2,5-dimethyl-			0.29	0.31
3.08	Pyridine		0.14	0.88	0.33
3.49	Toluene	2.59	2.17	2.10	2.18
4.17	3-Amino-s-triazole		0.40	0.41	0.46
5.07	Furfural	0.73	1.00	1.20	6.14
5.83	Benzene, 1,3-dimethyl-	0.47	0.53	0.49	0.53
6.07	p-Xylene	0.57	0.59	0.64	0.58
7.46	2(5H)-Furanone	0.45	0.52	0.32	0.32
7.80	1,2-Cyclopentanedione	0.18	0.88	0.54	0.65
9.51	Catechol	0.16	0.21	0.42	0.34
9.75	Phenol	1.70	1.43	1.48	2.25

9.99	Benzofuran	0.53	1.02	0.80	0.93

Table S2. The detailed results of PY-GC/MS test of samples at the RT range of 10-20 min.

RT/min	Compound	Peak area percentage (%)			
		CS	LS-M	LS-I	LS-O
10.4	Cyclotetrasiloxane, octamethyl-	1.94	1.07	1.59	1.67
11.05	1,2-Cyclopentanedione, 3-methyl-	0.46	0.56	0.60	0.55
11.5	Indene	1.47	1.05	0.81	0.96
11.99	Phenol, 2-methyl-	0.84	0.75	0.86	1.07
12.66	p-Cresol	0.81	1.51	1.43	1.99
12.98	Mequinol	1.61	3.04	2.41	1.56
13.66	Maltol		0.39		0.51
14.87	Phenol, 2,5-dimethyl-	0.40	0.71		0.68
15.45	Phenol, 3-ethyl-	0.26	0.38	0.39	0.54
15.7	Naphthalene	2.38	2.18	1.23	1.00
16.09	Creosol	1.39	3.10	1.15	0.62
16.41	Catechol	2.63	3.55	3.45	4.90
16.89	Benzofuran, 2,3-dihydro	0.57	0.40	0.37	0.41
17.24	5-Hydroxymethylfurfural	0.54	1.32	1.19	1.73
17.42	Phenol, 2-ethyl-5-methyl-	0.34	0.42		
18.10	1,2-Benzenediol, 3-methyl-	0.78	0.45	0.66	0.70
18.87	Naphthalene, 1-methyl-		0.44	0.37	0.33
18.94	1,2-Benzenediol, 4-methyl-	2.10	1.96	1.00	2.09

19.30	Naphthalene, 1-methyl-	0.38			
19.50	2-Methoxy-4-vinylphenol	1.81	0.34	1.68	1.20

Table S3. The detailed results of PY-GC/MS test of samples at the RT range of 20-30 min.

RT/min	Compound	Peak area percentage (%)			
		CS	LS-M	LS-I	LS-O
20.31	Resorcinol		0.26		
20.68	Eugenol	0.64	0.84	0.31	
21.42	1,3-Benzenediol, 4-ethyl-	0.69	0.47		0.62
21.74	Vanillin	1.48	1.89	0.87	1.22
22.0	trans-Isoeugenol	0.43	0.48		0.61
22.89	Biphenylene	2.0	1.42	0.44	0.48
23.96	4-Hydroxy-3-methoxyacetophenone	0.45	1.09	0.96	0.87
24.47	1-Naphthalenol	0.37	0.42		
25.06	2-Propanone, 1-(4-hydroxy-3-methoxyphenyl)-	0.55	0.90		
25.3	.beta.-D-Glucopyranose, 1,6-anhydr o-		0.72	1.13	2.46
25.88	Dodecanoic acid	0.68	0.32	0.82	0.94
26.06	Vanillic acid		0.73	0.40	0.53
26.67	Cedrol	1.19			
28.26	4-((1E)-3-Hydroxy-1-propenyl)-2-methoxyphenol	0.50	0.46		
29.61	Coniferyl aldehyde	2.07	1.51		0.37

29.72	4-((1E)-3-Hydroxy-1-propenyl)-2-methoxyphenol	4.29	0.28	0.82

Table S4. The detailed results of PY-GC/MS test of samples at the RT range of 30-40 min.

RT/min	Compound	Peak area percentage (%)			
		CS	LS-M	LS-I	LS-O
30.24	Tetradecanoic acid	0.32	0.27	0.28	0.37
34.4	n-Hexadecanoic acid	4.75	5.52	8.08	9.61
37.5	9,12-Octadecadienoic acid (Z,Z)-Linoelaidic acid	0.25	0.37	0.47	0.38
37.56	Oleic Acid	0.33	0.34	0.55	0.57
38.12	Octadecanoic acid	4.43	6.26	8.56	11.75
43.58	Squalene			9.40	8.86
